# Whole Body Cryotherapy and Hyperbaric Oxygen Treatment: New Biological Treatment of Depression? A Systematic Review

**DOI:** 10.3390/ph14060595

**Published:** 2021-06-21

**Authors:** Marek Krzystanek, Monika Romańczyk, Stanisław Surma, Agnieszka Koźmin-Burzyńska

**Affiliations:** 1 Clinic of Psychiatric Rehabilitation, Department of Psychiatry and Psychotherapy, Faculty of Medical Sciences, Medical University of Silesia in Katowice, Ziołowa 45/47, 40-635 Katowice, Poland; romanczykmonika@wp.pl (M.R.); stasiu.surma@onet.eu (S.S.); 2 Clinic of Psychiatric Rehabilitation, Leszek Giec Upper-Silesian Medical Centre of the Medical University of Silesia in Katowice, Ziołowa 45/47, 40-635 Katowice, Poland; salwadore@gmail.com

**Keywords:** whole body cryotherapy, WBC, hyperbaric oxygen treatment, HBOT, depression, pharmacotherapy, augmentation, treatment resistant depression

## Abstract

Treatment with antidepressants is often insufficiently effective, especially in treatment-resistant depression. In such a situation, it is possible to change the drug, add a second antidepressant, or use pharmacological and non-pharmacological methods of augmenting the effect of pharmacotherapy. New methods that may fall into the scope of multi-module depression treatment as an augmentation of depression treatment are whole body cryotherapy (WBC) and hyperbaric oxygen treatment (HBOT). 545 records were selected and analyzed for these two treatments and finally three clinical trials were selected for analysis. The review also includes data on the possibility of using WBC and HBOT in somatic indications and in organic mental syndromes. Despite the small number of studies on the effectiveness of WBC or HBOT in depression, the current data show that both methods may be effective in the treatment of depression. WBC may be effective in the augmentation of antidepressants, and additionally, it is a method in which a quick antidepressant effect is obtained. HBOT may be effective in endogenous depression, just as it is effective in the treatment of somatic depression symptoms. The results are very preliminary, but if confirmed in subsequent studies, both WBC and HBOT may become new treatment options in treating depression. The authors point to the need and directions for further research into these treatment methods as an augmentation strategy for pharmacological treatment of depression.

## 1. Introduction

Despite the progress in psychopharmacology and the introduction of new and effective antidepressants [1], the effectiveness of pharmacological treatment of depression is still far from satisfactory. Analyzes of the effectiveness of treating acute depressive episode on large groups of patients have shown that only about 1/3 of patients achieve remission after a 12-week treatment period [2] and most of those treated with even multiple drugs either do not achieve remission or it is not permanent [3,4]. Moreover, despite the efficacy of antidepressants confirmed in clinical trials [1], there is increasing concern about the slight difference between the effect of antidepressants and placebo.

A meta-analysis by Li et al. showed in patients pharmacologically treated for depression not only a high level of placebo response (SMD = 1.22, which indicates a very large therapeutic effect), but also an increase in the placebo effect in recent years [5]. One of the most recent meta-analyzes also indicates the limited efficacy of pharmacological treatment of depression in the elderly, for whom there is no statistically significant difference between treatment with an antidepressant and a placebo [6]. Moreover, the problem of ineffectiveness in treating depression increases with age. It was shown that the effectiveness of treating depression in the elderly population is two times lower than in people in early and middle adulthood, and amounts to 45.3% and 40.5% in the group of people treated with an antidepressant or a placebo, respectively [6].

Ineffective antidepressant treatment of at least two episodes of depression has been shown to lead to treatment-resistant depression (TRD), characterized by more frequent relapses with increasingly severe symptoms of depression, leading to chronic impairment of patients’ functioning and increased comorbidity, as well as suicide and non-suicidal mortality [4]. In the case of ineffective treatment of depression or TRD, treatment augmentation using biological and psychological methods of therapy is indicated.

Strategies to increase the effectiveness of treating depression include changing the antidepressant, adding another antidepressant, or augmenting the treatment by adding another drug like atypical antipsychotic or lithium. There are also non-pharmacological forms of augmentation of depression treatment that have been proven to be effective; they include: cognitive-behavioral psychotherapy, psychoeducation, aerobic exercise, neuromodulatory treatment through vagus nerve stimulation, electroconvulsive therapy (ECT), transcranial direct current stimulation (TDCS), repetitive transcranial magnetic stimulation (rTMS) or deep brain stimulation (DBS) and light therapy [3,4].

However, it turns out that non-pharmacological forms of biological treatment used in the treatment of TRD also do not show substantial efficacy. ECT, despite the improvement achieved during the electroshocks, has no lasting effect and even despite continued pharmacotherapy, relapses are observed in a large (37%) proportion of patients [7]. TDCS has so far been used with a moderate effect in depression, similarly to rTMS, which also has moderate and short-term efficacy in improving mood and cognitive functions in people with depression [1,7]. Light therapy has a proven, but low effectiveness in enhancing the antidepressant effect of antidepressants in both seasonal depression and recurrent depression [8].

Due to the incomplete effectiveness of various treatments for depression the current mainstream strategy in the treatment of TRD is the utilization of multi-module treatment, combining drugs, psychotherapy and non-pharmacological biological forms of treatment [4]. Moreover, insufficient effectiveness of pharmacological methods justifies the search for other, including biological and non-pharmacological methods of treating mental disorders that can be used as augmentation of pharmacological treatment. 

There are publications indicating the possibility of improving depressive symptoms on the basis of somatic diseases, treated with methods that use body stimulation with extreme physical stimuli—whole body cryotherapy (WBC) or hyperbaric oxygen treatment (HBOT), i.e., 100% oxygen at higher than atmospheric pressure [9,10,11]. Searching for new possibilities of augmentation of depression treatment, the authors decided to review the literature on the effectiveness of WBC and HBOT, which may be new methods of augmentation of pharmacological treatment of depression in multi-module treatment.

## 2. Materials and Methods

Looking for evidence that WBC and HBOT may be effective in treating depression authors decided to review the literature, considering clinical trials, as well as case series and case reports. PRISMA guidelines were used when preparing this systematic review [12]. The criteria for identification the study for the analysis were the presence of a diagnosis of depression and WBC or HBOT treatment. The analysis also included studies in which, apart from WBC or HBOT, a different treatment method was used. Only full-text publications available in English were considered in the analysis. In each study, at least the baseline and endpoints of treatment efficacy had to be characterized. After the screening, the analysis excluded experimental papers, review papers, clinical trials other than with depressive disorders, organic studies on depressive disorders and studies older than 20 years.

The following medical databases were searched in the study: PubMed, Scopus, Web of Science and Cochrane library. The following terms were used to search for clinical trials: “cryotherapy and depression”, “cryotherapy and depression and treatment”, “cryotherapy and psychiatry”, “hyperbaric oxygen and depression”, “hyperbaric oxygen and depression and treatment” and “hyperbaric oxygen and psychiatrists”. The search was performed according to the PICO framework (P—patient, problem or population, I—intervention, C—comparison, control or comparator, O—outcomes). During our search, we used the following terms: “bipolar depression” (Title/Abstract), “memantine” (Title/Abstract), “mood stabilizers” (Title/Abstract), “improvement of depressive symptoms” (Title/Abstract), “memantine and depression” (Title/Abstract); “memantine and depression treatment” (Title/Abstract), “monotherapy” (Title/Abstract) and “augmentation” (Title/Abstract). The study included publications up to 21/03/2021. The review was conducted independently by two investigators, then their search results were combined and duplicate records were removed. No study has been identified that investigated the effectiveness of hyperbaric oxygen in the treatment of endogenous depression, thus one study of depression comorbid with spinal cord injury was included in the analysis. 

**Figure 1:**
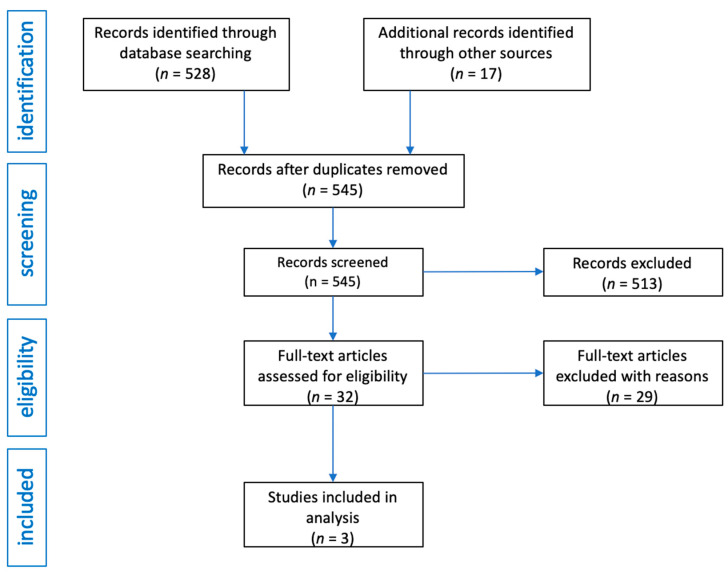
Flow diagram of studies analysis and selection for review.

After obtaining 528 records from the medical databases searched, the same terms were entered in the Google search engine and an additional 17 publications were obtained. In total, there were 545 records in the database of articles. After initial review, 513 articles were excluded as they were review articles, editorials, commentaries or letters to editor. Moreover, publications in which only abstracts were available, publications in a language other than English were also excluded. In this way, 32 articles were obtained, of which 29 were excluded because they were studies in which there was no basic data on group size, or inclusion and exclusion criteria, or when after reading it turned out they contain in-formation not related to the topic of the review. The flow diagram of the analysis is presented in Figure 1.

To assess the risk of bias and study quality in quantitative studies, the Effective Public Health Practice Project’s (EPHPP) Quality Assessment Tool for Quantitative Studies (QATQS) was used [13,14]. This tool enables quality evaluation of a wide range of study designs, including RCTs, observational studies with and without control groups and case studies. The instrument contains eight different sections, each with multiple questions: selection bias, study design, confounders, blinding, data collection methods, withdrawals and drop-outs, intervention integrity, and analyses. Each section receives a score of 1 (strong), 2 (moderate), or 3 (weak), and a final score is determined by the number of “weak” ratings. Strong rating is given to a study if there is no weak component score. Moderate rating is given with one weak component score. Weak rating is given with two or more component rating scores.

## 3. Results

Table 1 summarizes the data obtained from the analysis of publications on HBOT and WBC in the treatment of depression.

### 3.1. WBC

A randomized study by Rymaszewska et al. involved 60 outpatients suffering from depressive and anxiety disorders [15]. The study excluded people with circulatory or breathing insufficiency, clotting, embolism, inflammation of blood vessels, open wounds, ulcers, serious cognitive disturbances, fever, addictions, claustrophobia, and over-sensitivity to cold. The subjects were randomly assigned to two groups, 34 people were the control group, and 26 people were included in the study group. From the study group, 14 people were diagnosed with a depressive episode and 12 with an anxiety disorder (according to ICD-10). The majority of these people were women (84.6% in the study group). All participants had previously been treated with psychotropic drugs (in the study group: antidepressants (*n* = 20), beznzodiazepines (*n* = 14) and antipsychotics (*n* = 5)), which were not changed throughout the treatment with WBC).

The study group underwent 15 sessions of WBC in a cryogenic chamber (2–3 min, from −160 °C to −110 °C), held for 3 weeks from Monday to Friday. Depending on the diagnosis of depressive or anxiety disorders, the mental state was assessed respectively with 17-points The Hamilton’s depression rating scale (HDRS-17) and Hamilton’s anxiety rating scale (HARS) at the beginning (T1) and after each week of the study (T2, T3 and T4). A follow-up was carried out 3 and 6 months after the completion of the study. The study did not describe side effects and cases of people who did not complete the study. Either they were absent or it is not clearly stated.

In the group of patients treated with cryotherapy, a significant improvement in both depressive symptoms and anxiety symptoms was achieved after the 5th, 10th and 15th session as compared to the beginning of the treatment, and the improvement in each case was greater than in the control group. In the control group, a statistically significant improvement was observed after 3 weeks of the study, but this improvement was significantly smaller than in the study group.

The paper does not provide numbers relating to the severity of depressive symptoms in the following weeks. The values read from the published chart show that in HDRS-17, the intensity of symptoms in the study group decreased after one week (T2) by approx. 10% (approx. 3% in the control group), in T3 by approx. 16% (approx. 3% in the control group) and in T4 by approx. 35% (approx. 7% in the control group). The reduction in symptoms as compared to the start of the study (T1) was statistically significant in each case. As stated by the authors, a decrease of 50% from the baseline scores of depressive symptoms was observed in 34.6% (*n* = 9) of the study group and only in 2.9% (*n* = 1) of the control group. Follow-up data, however, were not provided in the study. According to the authors, their results indicate the possibility of using WBC as an add-on treatment in the treatment of both depression and anxiety disorders, however only for the short-term treatment [15].

In the second randomized and double-blind study from the same clinical site, conducted by Rymaszewska et al., the effectiveness of repeated short exposures to extremely low temperatures was assessed through the use of WBC on mood, quality of life, and biochemical parameters among outpatients with the diagnosis of depression who were treated pharmacologically [16]. The study involved 92 participants, aged 20–73 years, diagnosed with a depressive episode according to ICD-10.

The exclusion criteria were alcohol and drug abuse, dementia, inability to understand questions and written information, psychosis, suicidal thoughts, standard contraindications to use WBC (e.g., acute respiratory diseases, acute cardiovascular disease like coronary disease, circulatory insufficiency, unstable hypertension, cold intolerance, claustrophobia, cryoglobulinemia, cancer, deep vein diseases, hypothyroidism, neuropathies, purulent skin changes, Reynaud disease, pregnancy), and previous exposition to WBC treatment.

Subjects participating in the study were randomly assigned to two groups: the study group, in which WBC was applied with the temperature of −110 °C to 160 °C, or the control group, in which the low temperature of −50 °C was applied, that does not fall within the definition of cryotherapy. 34 people resigned from participation in the study before it started. The reasons were lack of time, claustrophobia, fear of WBC, or developing cold/flu. The entire study, i.e., 10 sessions over the course of 2 weeks, was completed by a total of 56 patients (30 from the study group and 26 from the control group), i.e., 61% of the patients recruited to the study.

The majority of this group were women (70%). The causes of drop-outs (*n* = 29) were hypertension, omitting more than two WBC sessions, developing a cold, getting burn with boiling water at home (non-related to the study) or unspecified in the study personal reasons. There were no serious somatic or adverse mental events during the treatment. All study and control group patients had been taking antidepressants (SSRIs or SNRIs) for at least 8 weeks (two subjects for at least 4 weeks) prior to study start and throughout the study period without any changes in the dosing. Initially, the patients in both groups did not differ significantly in the severity of depressive symptoms. As the primary measure, improvement of the mental state of patients was assessed using the Beck Depression Inventory-II (BDI-II) before the first WBC session (T1), then after the sixth (T2) and tenth session (T3), and 2 weeks after completion of WBC (T4). The psychiatrist assessed the severity of depression using the HDRS-17 on T1 and T4.

Comparing the mean severity of depression measured with the HDRS-17 scale, two weeks after the last WBC session (time point marked in the study as T4), the severity of depression decreased by 69% in the study group and by 42.6% in the reference group, while on the BDI-II scale the intensity of depressive symptoms in the study group decreased by 38% and in the reference group by 14.7%. These differences were significant both between T0 and T4 as well as between the groups at T4. Before T4, the researchers used only the BDI-II scale in the study. The results obtained therein indicate that the improvement in the study group was 56% in the T2 point (12.2% in the reference group), in T3 point—0% (14.6% in the reference group) and in T4—38% (14.7% in the reference group). The criterion of recovery at the T4 visit was met by 66.7% in the study group and 57.14% in the reference group (*p* = 0.55). Finally, the researchers concluded that WBC is an effective method supporting the pharmacological treatment of depression, and has a positive effect on the well-being and quality of life of people suffering from depression [16].

From the analyzed biochemical parameters, no significant changes in the level of interleukin 6 and 10 as well as high sesitivity C-reactive protein (hsCRP), nitric oxide (NO) and total antioxidant status (TAS) were observed after the end of WBC treatment. Importantly, the intent to treat analysis was not performed in both studies. The lack of this analysis makes it impossible to exclude type II error and limits the power of inference on the basis of the conducted analyzes.

### 3.2. HBOT

The aim of the randomized clinical trial by Feng et al. was to evaluate the effect of HBOT on mental problems in patients with incomplete spinal cord injury (ISCI), in particular depression and anxiety [17]. Sixty patients with ISCI participated in the study. Inclusion criteria were: (1) diagnosis of cervical, thoracic, or lumbar ISCI confirmed in by computed tomography (CT) or magnetic resonance imaging (MRI) according to the diagnostic criteria made by ASIA, (2) presence of depression and/or anxiety disorder based on Chinese Classification and Diagnostic Criteria of Mental Disorders, (3) mild to moderate severity of depressive episode (8–24 points of HAMD scale and/or 7–29 points of HAMA scale), (4) patients who received surgical treatment within 15 days after injury and had stable vital signs (5) ISCI caused by trauma and (5) patients aged 18 to 50 years. In turn, the criteria for exclusion from the study were as follows: (1) patients with craniocerebral injury or other nervous system diseases, (2) patients with cognitive dysfunction, (3) patients who had a history of psychiatric disorders, (4) ISCI caused by other causes except trauma and (5) patients who were not suitable for HBOT, such as untreated pneumothorax, mediastinal emphysema, pulmonary bulla, active bleeding, and tuberculous cavity combined with hemoptysis.

The patients in the study were randomized into three groups. In the first group, HBOT was additionally used (*n* = 20), and in the second group psychotherapy (*n* = 20) that was defined as supportive psychotherapy and cognitive behavioral therapy from psychiatrists and psychologists and comfort treatment from society and family. In the control group (*n* = 20), only routine conventional rehabilitation was used. Psychotherapeutic sessions and HBOT lasted for 8 weeks (once a day, 6 days a week). All groups were receiving routine rehabilitation therapy.

HBOT sessions were held in the HBOT chamber for multiple individuals. Patients were in decubitus position with oxygen mask or tracheotomy tube. The pressure in the HBOT chamber reached 0.2 MPa (2.0 ATA) at a uniform speed within 20 min, and lasted for 30 min twice. There was an interval of 10 min between the two 30-min HBOT, then the pressure in the HBOT chamber was uniformly reduced to normal pressure within 20 min. Each HBOT took 110 min. Before starting HBOT and after 8 weeks of treatment, depression and symptoms of anxiety were assessed using HDRS-24 and HAMA scales.

After 8 weeks of treatment, the HDRS-24 score decreased on average by 50% in the HBOT group and only by 12.5% in the control group. In turn, the symptoms of anxiety in HAMA scale in the HBOT group decreased by 48.2%, while in the control group by 31.2%. The differences in the severity of depression and anxiety between the HBOT group and the control group were statistically significant. Treatment results in the group treated with psychotherapy did not differ statistically from the HBOT group, therefore the authors conclude that the effect of HBOT on depression and anxiety is similar to psychotherapy [17]. According to the study report, all patients completed it. Based on the data available in the publication, no side effects occurred in patients treated with HBOT.

## 4. Discussion

WBC is a treatment method in which a special cryochamber is used, where the temperature is extremely low (between −110 °C and −160 °C). The patient stays there for a short time between 1–4 min. Exposures to low temperatures are repeated at different time intervals [18]. Scientific studies have shown, that the systematic use of extremely low temperatures reduces the level of IL-1α, and increases the level of cytokines IL-6 and IL-10, and also contributes to the reduction of the level of TAS, having an overall an immune-stimulatory effect [19,20]. So far, the effectiveness of WBC has been proven in the treatment of somatic diseases such as rheumatoid arthritis, multiple sclerosis, fibromyalgia, chronic back pain, and ankylosing spondylitis [18]. One study also showed that in patients with mild cognitive impairment (MCI), cryotherapy reduces the frequency of depressive symptoms measured with Visual Analogue Scales (10 sessions, study group *n* = 33, temperature from −110 °C to −160 °C) this justifies the attempt to use WBC to treat depression [9].

Our analysis of the publications shows a small number of studies on the effect of WBC on the treatment of endogenous depression. Only two published studies with the use of WBC as an add-on treatment for pharmacological treatment of patients diagnosed with depression were carried out so far, but they indicate that this method can bring about a quick improvement in patients who are already being treated pharmacologically. In both studies, this improvement is significant after 5–6 sessions of WBC. In the study with 10 WBC sessions, the intensity of depressive symptoms decreased by about 35% during this time, and in the study with 15 sessions, even by 69%. This may indicate a rapid and possibly dose-dependent treatment effect.

Conversely, there are some doubts about these results, because however in the study by Rymaszewska et al. patients were assessed psychiatrically with HDRS-17 every week, the depressed group was small (*n* = 14). In turn, in the next study by Rymaszewska et al. (2020) the group was larger, but the improvement in the following weeks was assessed using the subjective self-assessment scale BDI, and not in an objective psychiatric examining. Therefore, the conclusions regarding the speed of improvement drawn by the authors are only preliminary and require confirmation in studies with mental health assessment performed by a psychiatrist. Unfortunately, no data has yet been published on the follow-up and persistence of improvement due to WBC after the end of treatment. Definitely, the results on the effectiveness of WBC require replication in subsequent studies in much larger study groups—the study by Rymaszewska et al. (2008) included only a very small group of patients with depression. In both studies by Rymaszewska, women have the advantage in the study group, that raises the question of the effectiveness of WBC in the group of men. It is also worth emphasizing that the results of using HBOT in depression so far come only from one clinical center. Therefore, they need to be verified in a multi-center study of the effectiveness of this method in depression.

The experiences of using WBC in somatic medicine formed the basis for conducting WBC in depressive patients according to a protocol with numerous known contraindications, which limits the possibility of widespread use of this method. A study by Rymaszewska et al. from 2008 indicates 100% tolerance and no drop-outs, while the 2020 study was not completed by 36 people. This may indicate the possibility of a large number of side effects and the need for careful selection of people for treatment. Both studies published so far were outpatient studies. Perhaps conducting WBC in hospital treatment would allow for greater control of the patients’ clinical condition and might be associated with a lower risk of side effects.

Despite these doubts and questions, the demonstrated preliminary effectiveness of WBC in the augmentation of the treatment of depression is essential. Therefore, subject to careful patient selection, this method is a promising candidate for an effective method of augmentation of the pharmacological treatment of depression. It will be very interesting to study the effectiveness of WBC in a group of patients diagnosed with treatment-resistant depression. An open question is also the possibility of treating depression with WBC as monotherapy or in a multimodal treatment strategy in combination not only with pharmacotherapy but also with other non-pharmacological treatment methods and psychotherapy.

Research is also needed on the mechanism responsible for the improvement of the mental state of patients suffering from depression and treated with WBC, as it is so far unknown; it is possible that the mechanism of WBC is related to its previously demonstrated immune-modulatory effect [15,19,21]. Interestingly, in one of the studies analyzed, the level of IL-6 and IL-10 as well as TAS was not confirmed for the changes observed in the treatment of somatic patients [16]. Perhaps it is related to the disturbances in the immune system already existing in patients with depression, these results, however, require replication in subsequent studies of the effectiveness of WBC in depression. With regard to the mechanisms of action of WBC, in one of the previous studies, a statistically significant reduction in the brain-derived neurotrophic factor (*p* < 0.05) was observed among the analyzed biochemical parameters, however, this study was conducted not in depressed patients, but in MCI patients with depressive symptoms [9].

The second non-pharmacological augmentation method for depression treatment that we analyzed is HBOT. This method involves the use of 100% pure oxygen at a pressure above atmospheric pressure [21]. HBOT is widely used in patients with foot ulceration resulting from diabetes, with carbon monoxide poisoning, in patients with vascular dementia and after traumatic brain injuries [22,23,24,25]. A 2017 study by Lim et al. suggests that HBOT, in addition to reducing inflammation in the nervous tissue, may also inhibit serotonin reuptake, which may be beneficial in the treatment of depression [26]. This study encourages attempts to use HBOT in the treatment of depression, but as our analysis shows, there are hardly any such studies apart from the Feng et al. study we described [17].

Today it is already known, that HBOT is an effective and safe method of treating post-stroke depression—this topic has been the subject of numerous studies. In a recent meta-analysis of 27 clinical trials (17 randomized) by Liang et al. it was found that the use of HBOT is effective both as add-on treatment as well as in monotherapy and significantly reduces the severity of depression on the HAMD-17 scale (weighted mean difference (WMD) = −4.33; 95% CI (−4 82 to −3.84), *p* = 0.000) and the HAMD-24 scale (WMD = −4.31; 95% CI (−5.01 to −3.62), *p* = 0.000) [12]. It was also shown that HBOT caused fewer side effects compared to conventional antidepressant therapy (9.6% versus 16.6%). Based on the research carried out so far, the most common side effect in the group of patients treated with hyperbaric oxygen was ear pain [12].

The antidepressant effectiveness of HBOT was also demonstrated in a case study of a 45-year-old patient with depression in the course of Parkinson’s disease [13]. This patient did not agree to pharmacological treatment, because he did not believe that it could bring him any improvement. In addition, the man was burdened with a family history, his mother also suffered from Parkinson’s disease, diagnosed at the age of 40. Therefore, he underwent a 30-day HBOT, held in a hyperbaric chamber (the patient inhaled pure oxygen through the mask in 2 sessions of 40 min, separated by a 10-min break, pressure 2.0 ATA). His depressive condition was assessed before and after the intervention using HDRS. After 30-day therapy with hyperbaric oxygen, the intensity of depressive symptoms, measured on HDRS decreased by 20 points. One month after the end of treatment, an observation showed that the improvement in mood was maintained.

Both post-stroke depression and depression in the course of Parkinson’s disease are, however, examples of depression on an organic basis and the effectiveness of HBOT in organic mental syndromes is not a direct proof of the effectiveness of HBOT in endogenous depression. In our opinion, the partial evidence for the effectiveness of HBOT in depression may be the study by Feng et al., published 4 years ago, selected for our analysis. This study looks at depression associated with ISCI. Despite the ambiguities or possible methodological flaws, it is so far the only study available in the literature indicating the possibility of HBOT being effective in the treatment of depression that is not directly related to an organic cause.

Patients were qualified for the study after being examined by a psychiatrist and meeting the criteria for depression and/or anxiety disorder. Although the exclusion criterion is the lack of previous episodes of mental disorders, it does not mean that depression in the patients included in the study was associated with ISCI. For this reason, this study was included in the analysis, and was treated as a study of depression with accompanying somatic disease. The study indicates that the patients recruited for the study had depression with anxiety of a similar intensity, so there was no subset of patients with depression and anxiety disorders and the number of patients in the study group seems to be sufficient to draw conclusions from the study. Analysis of the results of this study prove that HBOT treatment can be effective in treating depressed patients, although the conclusion must be repeated in next studies where HBOT is used in depression without comorbidities.

Feng et al. state that HBOT treatment is as effective as psychotherapy, however, based on the methodology of the study it is difficult to define what type of psychotherapy was effective in the psychotherapy group [17]. However, the question remains, would the use of both methods at the same time be more effective and give a greater effect? The answer to this question requires another study comparing a clearly defined type of psychotherapy with HBOT used jointly and in monotherapy.

Although it may be assumed that since in the study by Feng et al. no side effects were described, they were not present, it seems rather unlikely and tolerance of HBOT treatment should be an element of assessment in subsequent studies. Some help, similarly to WBC, may be the experience with the use of HBOT in somatic indications. It may help rule out patients for whom this procedure is risky, thus reducing the risk of side effects.

## 5. Conclusions

**Figure 2:**
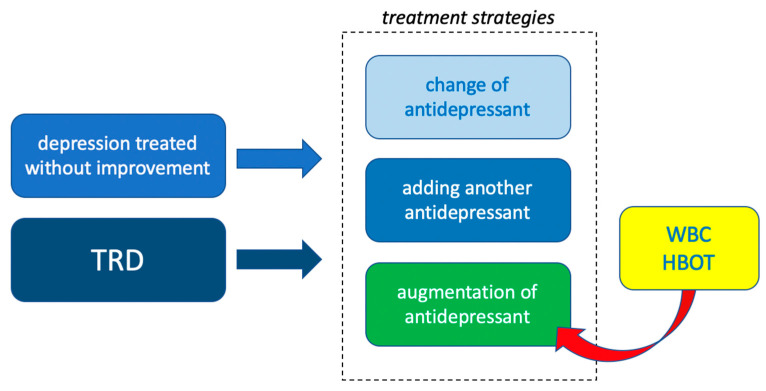
Whole Body Cryotherapy (WBC) and Hyperbaric Treatment (HBOT) could become new methods of antidepressant augmentation, also for treatment resistant depression (TRD).

The results obtained so far from the use of WBC in the augmentation of the treatment of depression are interesting, but require replication in subsequent studies. After confirming the effectiveness of the WBC, its high efficiency and quick treatment effect could make WBC an attractive method of augmentation of the effect of an antidepressant treatment possibly also in TRD. HBOT seems to be effective so far in treating post-stroke depression. The one pilot study conducted so far shows that HBOT may be highly effective in depression, but extensive research is needed to confirm its effectiveness in endogenous depression. Both methods may therefore be effective new ways to improve the effectiveness of treatment of a depressive episode as well as candidates for a treatment of TRD (Figure 2), however, it is too early to include them in treatment standards.

Both methods, WBC and HBOT, have been used in somatic medicine so far, so the protocols for these forms of therapy are well known and confirmed, which means that their introduction to psychiatry may be burdened with a lower risk of the frequency of drop-outs as well as side effects.

## Figures and Tables

**Table 1:** Summary description of the publications selected for the analysis, regarding influence of WBC or HBOT on depressive episode treatment. The risk of bias and study quality assessed with the Effective Public Health Practice Project’s Quality Assessment Tool for Quantitative Studies (QATQS) was presented as the global rating for each publication (1—strong, 2—moderate, 3—weak).

Authors	Year	Characteristics of Participants	Intervention	Results	Conclusions	QATQS Global Rating
**WBC**						
Rymaszewska et al. [15]	2008	Adults aged 18–65 years with a diagnosis of depression and anxiety disorders	60 participants took part in the study. The control group consisted of 34 people, and the study group was 26 people. Both groups received psychopharmacotherapy, in addition, the study group underwent 15 sessions of WBC	3 weeks after the WBC treatment, the HDRS-17 score was reduced by at least 50% in 34.6% of the study group	WBC may be an effective adjunct to treating depression and anxiety disorders in the short-term therapy	2
Rymaszewska et al. [16]	2020	Adults (aged 20–73) diagnosed with a depressive episode, medically stable.	92 participants were recruited into the study and 56 completed. The participants were divided into 2 groups—experimental one (*n* = 30) with WBC treatment and control group (*n* = 26) in which low temperature (−50 °C) was used. The subjects completed 10 sessions	Statistically significant reduction in the intensity of depression symptoms assessed in the HAMD-17 and BDI-II scales.	WBC can be an effective treatment for depression.	1
**HBOT**						
Feng et al. [17]	2017	Adults aged 18–50 years with incomplete spinal cord injury in the cervical, thoracic or lumbar spine, with comorbid depression and anxiety.	60 people participated in the study. They were divided into 3 groups. In the first one, HBOT was used (*n* = 20), in the next—psychotherapy (*n* = 20). In the control group (*n* = 20), only conventional rehabilitation was used. Psychotherapeutic sessions and HBOT lasted for 8 weeks (once a day, 6 days a week).	After 8 weeks of treatment, the HAMD-24 score was significantly lower both in the group treated with HBOT and in the group participating in psychotherapeutic sessions, compared to the control group (*p* < 0.05).	HBOT and psychotherapy have a similar effect on depression and anxiety.	2

## Data Availability

No new data were created or analyzed in this study. Data sharing is not applicable to this article.
